# Urban–rural differences in immunisation status and associated demographic factors among children 12-59 months in a southwestern state, Nigeria

**DOI:** 10.1371/journal.pone.0206086

**Published:** 2018-11-05

**Authors:** Ibidolapo T. Ijarotimi, Akinola A. Fatiregun, Oluwapelumi A. Adebiyi, Olayinka S. Ilesanmi, Olufemi Ajumobi

**Affiliations:** 1 Nigeria Field Epidemiology and Laboratory Training Program, Asokoro, Abuja, Nigeria; 2 Ondo State Office, World Health Organization, Akure, Ondo State, Nigeria; 3 Department of Community Medicine, University College Hospital, Ibadan, Nigeria; The University of Warwick, UNITED KINGDOM

## Abstract

Vaccine preventable diseases (VPDs) are a leading course of child under-five mortality in sub-Saharan Africa. A target of 95% immunization coverage is necessary for the sustained control of VPDs. This study aims to determine the immunization status and its associated demo-graphic factors among children 12–59 months old in Akinyele Local Government area (LGA), Oyo State, Nigeria. A community-based cross-sectional study was carried out in one urban and one rural ward of Akinyele LGA. Fourhundred and forty-four (449) Under-five children were selected by multistage sampling technique. Data were collected from caregivers using interviewer administered questionnaires. Odds ratios at 95% CIand Chi square at 5% significant level were computed to identify the factors associated with non or partial immunisation. Multiple logistics regression at 5% significance level was done to determine the socio-demographic determinants of immunisation status. Overall, 449 children aged 12–59 months were surveyed of which 213(47.4%) were males and 236(52.6%) were from urban area. Overall, 365(81.3%) was fully immunized, 75(16.7%) was partially immunized and 9(2.0%) had never been immunized. Predictors of a child being partially or un-immunised were being in the fourth wealth quintile (AOR 7.9; 95%CI: 2.7–18.0), poorest wealth quintile (AOR 14.5; 95%CI 4.2–20.5), having a mother with no education (AOR 6.4; 95%CI: 2.9–14.1) and a mother that practiced Islam (AOR: 2.2; 95%CI: 1.3–3.7). Immunisation coverage was somewhat high but still suboptimal among the study population. Strategies that improve female literacy and those that target religious institutions may be effective in improving immunisation uptake.

## Introduction

Childhood immunization has proved to be the most important child survival strategy. Estimated to prevent between 2 and 3 million deaths each year [1], it is one of the most essential and cost-effective strategies to reduce childhood morbidity and mortality [2]. It is also one of the key elements of primary health care [3].

According to WHO estimation in 2008, 1.5 million deaths or 17% of global mortality in children under 5 years were due to only 6 Vaccine Preventable Diseases (VPDs) [4]. Despite the established health and economic benefits of childhood vaccinations, [5] child vaccination coverage, especially in developing countries, is still low VPDs continue to be a leading course of child under-five mortality in sub-Saharan Africa [6]. In 2016, an estimated 19.5 million infants did not have access to routine immunisation services globally. About 60% of these children, live in 10 countries: Angola, Brazil, the Democratic Republic of the Congo, Ethiopia, India, Indonesia, Iraq, Nigeria, Pakistan and South Africa [1].

Initially, Nigeria, with the Expanded Program on Immunisation (EPI), made significant progress towards achieving universal immunisation coverage with the coverage rate, the highest ever documented, reaching 81.5% in the early 1990s [7]. Unfortunately, this progress was short-lived and despite introduction of supplemental immunization programs and various strategies, low and fluctuating levels of immunization coverage remain a significant public health problem in poorer areas of Nigeria [7]. By 2003, immunization coverage was documented to be as low as 12.9% for all antigens [8]and in 2012, in certain LGAs of the country, coverage levels as low as 3% were recorded [7]. The most recent Nigeria Multiple Indicators Survey (MICS) carried out in 2016-17revealed that only 21% of children 12–23 months and 18.8% of children 24–35 months were fully immunised [9].

While the standard measure of vaccination coverage is the percentage of children who have received the requisite number of vaccine doses irrespective of the age at receipt of the vaccine [9], toensure maximum protection against vaccine-preventable diseases, a child should receive all immunizations within recommended intervals. According to the World Health Organisation (WHO), in order to benefit from the potential population-wide benefits of routine vaccination through herd immunity, a national target of 95% coverage annually for each antigen in the routine immunisation schedule must be achieved by 2 years of age [10].

Before the addition of newer vaccines like Heamophilus Influenza b (Hib) and Pneumococcal Conjugate Vaccine (PCV) to the national immunisation schedule, a child was considered fully immunized if he or she had received a BCG vaccination against tuberculosis; three doses of DPT to prevent diphtheria, pertussis (whooping cough, and tetanus; at least three doses of polio vaccine; at least three doses of Hepatitis B and one dose of measles vaccine. All these vaccinations should be received during the first year of life, over the course of five visits [11].

Studies conducted in Nigeriaidentifiedsome socio-demographicfactors associated with incomplete or non immunisation of children. These include maternal age [12], location (urban versus rural) [11], parental education [13, 14]socioeconomic factors [12, 13] knowledge of immunisation [14, 15] religion and socioeconomic status [12]. In addition, the 2016–17 MICS and previous Nigeria Demographic and Health surveys (NDHS)documented wide gaps in immunization coverage related to maternal factors like age, educational status and wealth quintiles and also location and tribe [9,16,17].

There is an urgent need to address specifically and concretely the problem facing immunisation activities in the country. Knowledge of socio-demographic barriers to effective immunisation programs is very important in the development and implementation of appropriate solutions. This study therefore aimed to determine the immunisation status and associated demographic factors among under-5 children and their caregivers in Akinyele Local Government Oyo State, Nigeria.

## Materials and methods

### Study area

A quantitative community-based cross-sectional study was conducted within the rural and urban communities of Akinyele Local Government Area (LGA) of Oyo State. Oyo state is one of the 36 states in the Southwestern region of the country. Akinyele LGA consists of a small urban part around a large number of rural settlements. Akinyele Local Government Area had two tertiary health facilities, two state-owned health facilities 32 primary health centres/maternity center and 38 registered private health institutions. The Local Government itself hadthe following healthcare workers in its employment; one medical doctor, 21 nurses/midwives and 101 other cadres of health workers. According to the 2013 NDHS survey only 25.8% of children 12–23 months had received basic immunisation defined as BCG, measles, and 3 doses each of DPT and polio vaccine (excluding polio vaccine given at birth) [9]. The study population were children 12–59 months old and their mothers/primary care givers living in the rural and urban communities of Akinyele LGA Ibadan. Children with severe immune-compromise and those with established allergies to vaccines were excluded.

### Sample size and sampling

We determined the number of children to be sampled using the effective sample size (ESS) by expected coverage and desired precision for 95% confidence interval (CI) according to the WHO [18]. Using an expected coverage of 42.8% in the Southwest [16], we obtained a minimum sample size of 410 children. A multistage sampling technique was employed to obtain a representative sample of children 12–59 months.

Stage 1: Akinyele Local Government Area was randomly selected by balloting from a list of Local Governments that had both urban and rural communities in Oyo state.
Stage 2: All the wards in Akinyele Local Government Area were then stratified into two groups- rural and urban. Ijaiye, a rural ward andOjoo, an urban ward were selected by balloting.
Stage 3: A sampling frame of all the communities in the two wards was drawn and 15 communities were selected from each ward by simple random sampling.
Stage 5: All houses in the selected communities were visited, (in houses that have more than one household, the household that was included was determined by balloting) and one child per household was selected by balloting.

### Operational definitions

Vaccination status of children: Based on the type and doses of valid RI antigens received, we categorized the children as fully immunized, partially immunized, or un-immunized. We defined these categories of vaccination status as follows:—Fully immunized child: a child who had received one dose of BCG, three doses of OPV (excluding OPV given at birth), three doses of DPT vaccine and one dose of measles vaccine (MCV1) by 12 months of age—Partially immunized child: a child who missed at least any one of the above doses;—Un-immunized child: a child who had not received any vaccine by 12 months of age [8,9]. We considered a child’s BCG vaccine valid, if a scar was present irrespective of whether the vaccination was recorded on the card or obtained by history. BCG vaccination recorded on the card but without a scar was also considered valid.

### Data collection

Data were collected using interviewer administered questionnaires with both open and closed ended questions. The questionnaires were administered to primary caregivers of selected children. The questionnaire was adapted from the UNICEF Multiple Indicator Cluster Surveys and Nigeria National Demographic and Health Survey Questionnaires. The questionnaire was pretested at Egbeda Local Government also in Oyo State.

Information was collected from the respondents under the following sections:

Section A: Demographic data

This included 21 questions which was used to obtain information on the child’s age, sex and birth order and also on the parent’s age, educational status, religion, income and occupation. Information on the number of under five children in the household was also obtained.

Section B: Household Wealth and characteristics

This consisted of 27 questions and assessed the socioeconomic status of the parents and their household wealth and characteristics.

Section C: Immunisation History

This consisted of 23 questions which assessed the immunisation status of the children. Data was collected from mothers to determine the vaccination status of the children in addition to making use of the children’s vaccination cards where available.

### Data processing and analysis

Quantitative data collected were checked, cleaned and entered into SPSS version 21. Parents’ socioeconomic status was assessed by using the principal components analysis of household assets and characteristics. The household assets used were ownership of car, refrigerator, stove, clock, mobile phone, television, and fan, radio, mobile telephone, washing machine, microwave, generator, computer/laptop, air conditioner, cable television, electricity, cooking stove. The household characteristics used were source of drinking water, waste disposal and roofing, flooring and wall materials. Odds ratiosat a 95% confidence interval and Chi square at 5% significant levelwere calculated to determine the factors associated with calculated immunisation status. Multiple logistics regression, at5% significant level was done to determine the predictors of non or partial immunisation.

### Ethical considerations

Written ethical approval for this study was givenbyOyo State Ethical Review Board (Reference number: AD/13/479/346). Permission to conduct the study was obtained from the Local Government Chairman, community leaders and heads of households. Written informed consent was sought from the primary care givers; they were informed of their right to decline or withdraw from the study at any time without any adverse consequences.

## Results

### Socio-demographic characteristics of parents/caregivers

A total of 452 mother-child pairs were approached and 449 responded giving a response rate of 99.3%. Of the 449 respondents, 236 (52.6%) were from urban areas of the LGA. The overall mean age of the mothers was 31.0± 4.4 years. The mean age of mothers from urban areas, 31.8 ± 4.5 years, was higher than that of mothers from rural areas; 30.2 ± 4.2 years (p<0.0001). A higher number of mothers from urban areas completed secondary school education 160 (67.8%), 59 (25.0%) completed tertiary school and only 2 (0.8%) had no formal education while 116(54.5%) of mothers from rural areas completed only primary education, 61(28.6%) completed only secondary education and 34 (16.0%) had no formal education (p<0.001). There was a significant difference in educational attainment among fathers between the urban and rural areas with 141(59.7%) having completed secondary education and 89(37.7%) having completed tertiary education in urban area, while only 98 (46.0%) completed secondary education and 4(1.9%) completed tertiary education among fathers in the rural area (p-<0.001). There was a significant difference in the distribution of the urban and rural families into wealth quintiles. Seventy-five (31.8%) of urban families were in the highest wealth quintile while none was in the lowest quintile. In the rural area, only 1 (0.5%) family was from the highest wealth quintile while 91 (42.7%) were from the lowest wealth quintile (p<0.001). Table 1.

**Table 1:** Socio-demographic characteristics of parents by location, Akinyele LGA, Oyo State, Nigeria—2013.

Characteristics	Urban N = 236	Rural N = 213	Total N = 449	Chi^2^ *p-*value
	n (%)	n (%)	n (%)	
**Mothers age group (years)**				
18–25	12 (5.1)	20 (9.4)	32(7.1)	
26–35	174 (73.7)	172 (80.8)	346 (77.1)	**0.002**
≥ 36	50 (21.1)	21 (9.9)	71 (15.8)	
**Mother’s religion**				
Christianity	167 (70.8)	136 (63.8)	303(67.5)	0.118
Islam	69 (29.2)	77 (36.2)	146 (32.5)	
**Wealth quintiles**				
Lowest	0 (0.0)	91 (42.7)	91 (20.3)	
2^nd^	4 (1.7)	80 (37.6)	84 (15.7)	
3^rd^	41 (17.4)	40 (18.8)	81 (18.0)	**<0.001**
4^th^	116 (49.2)	1 (0.5)	117 (26.1)	
Highest	75 (31.8)	1 (0.5)	76 (16.9)	
**Type of marriage**				
Single mother	7 (3.0)	4 (1.9)	11 (2.4)	
Monogamous	217(91.9)	186(87.3)	403 (89.8)	0.064
Polygamous	12(5.1)	23 (10.8)	35 (7.8)	
**Mother’s ethnicity**				
Yoruba	222(94.1)	187 (87.8)	409 (91.1)	**0.020**
Others^#^	14 (5.9)	26(12.2)	40 (8.9)	
**Mother’s highest educational level attained**				
None	2 (0.8)	34 (16.0)	36 (8.0)	
Primary school	15(6.4)	116 (54.5)	131 (29.2)	**<0.001**
Secondary school	160(67.8)	61 (28.6)	221 (49.2)	
Tertiary school	59 (25.0)	2 (0.9)	61 (13.6)	
**Father’s religion**				
Christianity	150 (63.6)	119(55.9)	269 (59.9)	0.097
Islam	86(36.4)	94 (44.1)	180 (40.1)	
**Father’s ethnicity**				
Yoruba	150(63.6)	119(55.9)	269 (59.9)	0.097
Others^#^	86(36.4)	94(44.1)	180 (40.1)	
**Father’s highest educational level attained**				
None	2 (0.8)	23(10.8)	25 (5.6)	
Primary school	4(1.7)	88(41.3)	92 (20.5)	**<0.001**
Secondary school	141(59.7)	98(46.0)	239 (53.2)	
Tertiary school	89(37.7)	4(1.9)	93 (20.7)	

^#^Ibo, Hausa, Igede, Tiv, Ewe (Togolese), Akan (Ghanaian).

Among the children, their mean age was 26.7± 18.1 months. The mean age of the children from urban location was 33.2 ± 18.2 months while that of the children from rural location was 25.8 ±18.0 months (p = 0.220). Age group 12–23 months, constituted the most among both urban 70 (29.7%) and rural, 60 (28.2%) respondents (p = 0.766). The majority, 126 (53.4%), of the children from urban area were males; while majority, 226(50.3%), of those from rural areas were females (p = 0.097). Table 2.

**Table 2:** Demographic characteristics of the children by location, Akinyele LGA, 2013.

Variables	Urban N = 236 n (%)	Rural N = 213 n (%)	Total N = 449 n (%)	Chi2 p-value
**Sex**				
Male	126 (53.4)	97 (45.5)	223 (49.7)	0.097
Female	110 (46.6)	116 (54.5)	226 (50.3)	
**Age in months**				
12–23	70 (29.7)	60 (28.2)	130 (29.0)	
24–35	58 (24.6)	53 (24.9)	111 (24.7)	0.766
36–47	54 (22.9)	57 (26.8)	111 (24.7)	
48–59	54 (22.9)	43 (20.2)	97 (21.6)	

### Immunisation status

Of 449 children, a total of 365 (81.3%) were fully immunised, 75 (16.7%) were partially immunized and 9(2.0%) were un-immunised. Of those that were immunised, 424 (96.4%) mothers claimed to have their children’s immunization cards but the cards were only seen for 45(10.6%) of these. The immunisation coverage for each antigen except Measles and Yellow fever was over 95% in the urban area. In the rural area only OPV 0 coverage was over 95%. Overall, antigens given at birth(BCG, OPV0, and Hep1)had a coverage of over 95%. Table 3.

**Table 3:** Distribution of children who received each antigen by location, Akinyele LGA, Oyo State, Nigeria– 2013.

Antigen dose	Number of children received		
	Urban N = 236 n(%)	Rural N = 213 n(%)	Total N = 449 n (%)
**BCG**	236(100)	204(95.8)	440 (98.0)
**OPV**			
0	236 (100)	203 (95.3)	439 (97.8)
1	235 (99.6)	178 (83.6)	413 (92.0)
2	230 (97.5)	169 (79.3)	399 (88.9)
3	230 (97.5)	169 (79.3)	399 (88.9)
**DPT**			
1	235 (99.6)	181 (85.0)	416 (92.7)
2	229 (97.0)	169 (79.3)	398 (88.6)
3	229 (97.0)	168 (78.9)	397 (88.4)
**Hepatitis**			
1	236 (100)	196 (92.0)	432 (96.2)
2	233 (98.7)	176 (82.6)	409 (91.1)
3	229 (97.0)	168 (78.9)	397 (88.4)
**Measles (MCV1)**	223 (94.5)	145 (68.1)	368 (83.0)
**Yellow Fever**	222 (94.1)	145 (68.1)	367 (81.7)

On bi-variate analysis, children in rural area were more likely to be partially or un-immunised compared to children in urban area (OR:6.4; 95%CI: 3.7–11.6). Children of the 4th or higher birth order were more likely to be partially or un-immunised than 1st born children (OR:3.4; CI: 1.5–7.8). Children in the fourth (OR-9.4; 95%CI 2.7–12.4) and the poorest quintile (OR- 18.3; 95% CI: 5.4–22.3) were more likely to be partially or un-immunised compared with children in the highest wealth quintile. Children in polygamous homes were also more likely to be partially or un-immunised (OR:2.8; CI: 1.3–5.8) compared to children in monogamous or single mother homes. Children of mothers that completed no education (OR 19.1; 95% CI: 10.5–26.4), primary education (OR: 12.5; 95% CI: 4.2–19.9) or secondary education (O: 3.6; 95% CI: 1.2–10.4) were all more likely to be partially or un-immunised compared to children of mothers that completed tertiary education. Children of fathers that completed no education (OR 24.1; 95% CI 18.8–36.0), primary education (OR 13.0; 95% CI 4.4–18.8) or secondary education (OR 3.6; 95% CI 1.2–10.34) were all more likely to be partially or un-immunised compared to children of fathers that completed tertiary education. Children born to mothers that practiced Islam were more likely to be partially or un-immunised compared to children born to Christian mothers (OR:2.4;95% CI: 1.5–3.8), similarly, children born to Muslim fathers were twice more likely to be partially or un-immunised compared to those born to Christian fathers (OR:2.1; 95% CI: 1.3–3.4).

However, on logistic regression, being in the fourth wealth quintile (OR 7.9; 95% CI 2.7–18.0), poorest wealth quintile (OR: 14.5; 95% CI: 8.2–20.5), having a mother with no education (OR 6.4; 95% CI 2.9–14.0) and a mother that practiced Islam (OR 2.2; 95% CI 1.3–3.7) were the only predictors of a child being partially or un-immunised. Table 4.

**Table 4:** Socio-demographic predictors of immunisation status, Akinyele LGA, Oyo State, Nigeria—2013.

Socio-demographic characteristics	Partially or un-immunised	OR (95%CI)	AOR (95% CI)
	N = 84		
	n (%)		
**Location**			
Urban	16 (6.8)	**Ref**	
Rural	68(31.9)	**6.4 (3.7–11.6)**	0.98 (0.30–3.13)
**Sex**			
Male	39 (17.5)	Ref	
Female	45 (19.9)	1.2 (0.7–1.9)	
**Child’s birth order**			
1st	9 (10.7)	**Ref**	
2nd or 3rd	41 (48.8)	1.2 (0.5–2.5)	0.8 (0.3–1.9)
4th or higher	34 (40.5)	**3.4 (1.5–7.8)**	1.9 (0.8–4.8)
**Wealth quintiles**			
Richest	3 (3.6)	**Ref**	
Second	7 (8.3)	1.6 (0.4–6.2)	1.5 (0.4–6.2)
Middle	12 (14.3)	**4.2 (1.1–16.5)**	2.7 (0.7–10.4)
Fourth	23 (27.4)	**9.2 (2.7–32.4)**	**7.9 (4.7–18.0)**
Poorest	39 (46.4)	**18.3 (5.4–62.3)**	**14.5 (8.2–20.5)**
**Family type**			
Monogamous/ Single mother	71 (84.5)	**Ref**	
Polygamous	13 (15.5)	**2.78(1.3–5.8)**	1.4 (0.6–3.3)
**Mother’s educational level competed**			
Tertiary	2 (2.4)	**Ref**	Ref
Secondary	22 (26.2)	**3.6 (1.2–10.4)**	1.8 (0.3–9.8)
Primary	42 (50.0)	**12.5 (4.2–19.9)**	4.2 (0.7–26.2)
None	18 (21.4)	**19.1 (10.5–26.4)**	**6.4 (2.9–14.0)**
**Father’s educational level completed**			
Tertiary	4 (4.8)	**Ref**	
Secondary	33 (39.2)	**3.6(1.2–10.4)**	1.2 (0.3–4.9)
Primary	34 (40.5)	**13.0 (4.4–18.8)**	1.3 (0.3–6.3)
None	13 (15.5)	**24.1(18.8–36.0)**	1.9 (0.4–11.6)
**Mother’s religion**			
Christianity	43 (51.2)	**Ref**	
Islam	41 (48.8)	**2.4 (1.5–3.8)**	**2.2 (1.3–3.7)**
**Father’s religion**		
Christianity	38 (43.2)	**Ref**	
Islam	46 (54.8)	**2.1 (1.3–3.4)**	0.9 (0.4–2.4)

## Discussion

We found thatthat though children living in rural areas were more likely to be partially or un-immunised, location was not a predictor of immunisation status. Different studies and the NDHS have documented higher immunisation coverages in the urban areascompared with the rural areas [9,16,17,19], however the only two studies found that assessed location as a predictor of immunisation gave conflicting findings. The study by Adedokun et al. which made use of a national data found that location was a predictor of immunisation status [12] while that of Olugbenga-Bello et al in North Central Nigeria did not find a relationship between location and immunisation status [20]. In view of this contrasting findings there is need for further research into the influence of location on immunisation status of children.

This study found the socio-demographic predictors of immunisation status to be maternal education, maternal religion and the family’s wealth quintile. This is similar to the findings of the study by Antai, in which he made use of2003 NDHS data [21], the study by Fatiregun and Okoro in Southern Nigeria [22]and the study by Oleribe et al which also made use of 2013 NDHS data [13). In addition to maternal factors, the study by Oleribe also found paternal literacy to be associated with immunisation status [13]. While our study did find a strong association between paternal literacyand immunisation status it did not find paternal literacy to be a predictor. This suggests there may be other intervening or confounding variables. One of such is probably the wealth index. Wealth index is a predictor of immunisation status. A higher education will often translate to a better job for the father and a higher income for the family. Another of such variables may also be the maternal literacy. Maternal literacy, as noted earlier is a predictor of immunisation status and it is also related to paternal literacy [23].

BCG coverage was quite high compared to the national figures but comparable to figures from Southwest Nigeria [9]. NDHS data have consistently shown that immunisation coverage is higher in Southwest Nigeria though the values were lower than that gotten by this study [9, 16]. It may this high because BCG was given at birth and as far as a woman delivers in the hospital, the child will likely get BCG vaccine before being discharged home [12].

Coverage with the first dose of measles vaccine can be used to measure the ability of health services to vaccinate children beyond early infancy. Also in addition, 95% coverage is required to prevent measles epidemics [18]. However, similar to this study, other studies have found varying (15%-79.5%) but suboptimal measles immunisation coverage in Nigeria [11, 14, 21]. These suggests that the health services are under-performing in their ability to vaccinate children beyond early infancy. While the National Supplemental Immunisation Activities (SIAs) may close the gap, the country still records regular outbreaks of measles [24] meaning that despite this additional strategy, measles immunisation coverage still remains lower than 95%.

There are major limitations to our study that may affect the interpretation of the results and the generalisation of the findings. Immunisation rates have consistently varied widely across geopolitical zones of the country [10, 16] so our results may not be generalisable to other zones. Another limitation of this study was that it was conducted among children 12–59 months in contrast to the recommended 12–23 months. Even when studies are limited to children aged 23 months, recall bias could be an issue. Recall bias will likely be worse in this study given the inclusion of children up to 59 months. It is however noteworthy that in addition, the number of vaccination cards seen and hence the immunisation history that could be verified was limited therefore the immunisation coverage could actually be much lower than that gotten by this study thoughstudies have shown that maternal recall is usually highly sensitive [25]. Lastly, this study did not put into consideration the timeliness of the vaccine received. If a child received a vaccine much later than he should have according to the schedule, he was still classified as having received the vaccine.

## Conclusion

Immunisation coverage was somewhat high but still suboptimal among the study population. Maternal factors influence immunisation status. Though location and paternal factors are strongly associated with immunisation status, their effect is due to other confounding or intervening factors. There is need for further research into this. However, presently strategies that improve female literacy and those that target religious institutions may be effective in improving immunisation uptake.

## Supporting information

S1 Data Immunisation status among children 12–59 months in a southwestern state, Nigeria. (XLSX)
